# Binocular head-mounted chromatic pupillometry can detect structural and functional loss in glaucoma

**DOI:** 10.3389/fnins.2023.1187619

**Published:** 2023-06-29

**Authors:** Yadan Quan, Huiyu Duan, Zongyi Zhan, Yuening Shen, Rui Lin, Tingting Liu, Ting Zhang, Jihong Wu, Jing Huang, Guangtao Zhai, Xuefei Song, Yixiong Zhou, Xinghuai Sun

**Affiliations:** ^1^Department of Ophthalmology and Visual Science, Shanghai Medical College, Eye, Ear, Nose and Throat Hospital, Fudan University, Shanghai, China; ^2^Shanghai Key Laboratory of Visual Impairment and Restoration, Fudan University, Shanghai, China; ^3^NHC and Chinese Academy of Medical Sciences Key Laboratory of Myopia, Fudan University, Shanghai, China; ^4^Institute of Image Communication and Network Engineering, Shanghai Jiao Tong University, Shanghai, China; ^5^Department of Retinal Disease, Shenzhen Eye Institute, Shenzhen Eye Hospital, Shenzhen Eye Hospital Affiliated to Jinan University, Shenzhen, China; ^6^Department of Ophthalmology, Ninth People's Hospital of Shanghai, Shanghai Jiao Tong University School of Medicine, Shanghai, China; ^7^Shanghai Key Laboratory of Orbital Diseases and Ocular Oncology, Shanghai, China; ^8^State Key Laboratory of Medical Neurobiology, Institutes of Brain Science and Collaborative Innovation Center for Brain Science, Fudan University, Shanghai, China

**Keywords:** pupil light response (PLR), pupillometry, melanopsin, optic nerve (ON), glaucoma, chromatic light

## Abstract

**Aim:**

The aim of this study is to evaluate the utility of binocular chromatic pupillometry in detecting impaired pupillary light response (PLR) in patients with primary open-angle glaucoma (POAG) and to assess the feasibility of using binocular chromatic pupillometer in opportunistic POAG diagnosis in community-based or telemedicine-based services.

**Methods:**

In this prospective, cross-sectional study, 74 patients with POAG and 23 healthy controls were enrolled. All participants underwent comprehensive ophthalmologic examinations including optical coherence tomography (OCT) and standard automated perimetry (SAP). The PLR tests included sequential tests of full-field chromatic stimuli weighted by rods, intrinsically photosensitive retinal ganglion cells (ipRGCs), and cones (Experiment 1), as well as alternating chromatic light flash-induced relative afferent pupillary defect (RAPD) test (Experiment 2). In Experiment 1, the constricting amplitude, velocity, and time to maximum constriction/dilation were calculated in three cell type-weighted responses, and the post-illumination response of ipRGC-weighted response was evaluated. In Experiment 2, infrared pupillary asymmetry (IPA) amplitude and anisocoria duration induced by intermittent blue or red light flashes were calculated.

**Results:**

In Experiment 1, the PLR of POAG patients was significantly reduced in all conditions, reflecting the defect in photoreception through rods, cones, and ipRGCs. The variable with the highest area under the receiver operating characteristic curve (AUC) was time to max dilation under ipRGC-weighted stimulus, followed by the constriction amplitude under cone-weighted stimulus and the constriction amplitude response to ipRGC-weighted stimuli. The impaired PLR features were associated with greater visual field loss, thinner retinal nerve fiber layer (RNFL) thickness, and cupping of the optic disk. In Experiment 2, IPA and anisocoria duration induced by intermittent blue or red light flashes were significantly greater in participants with POAG than in controls. IPA and anisocoria duration had good diagnostic value, correlating with the inter-eye asymmetry of visual field loss.

**Conclusion:**

We demonstrate that binocular chromatic pupillometry could potentially serve as an objective clinical tool for opportunistic glaucoma diagnosis in community-based or telemedicine-based services. Binocular chromatic pupillometry allows an accurate, objective, and rapid assessment of retinal structural impairment and functional loss in glaucomatous eyes of different severity levels.

## 1. Introduction

Glaucoma is the most frequent cause of irreversible blindness worldwide and is characterized by a decline in retinal ganglion cells (RGCs) and visual function damage (Jonas et al., 2017). By 2020, among the global population with moderate or severe vision impairment, the number of people affected by glaucoma was ~4.5 million. Among the global population with blindness, the number of patients with glaucoma is anticipated to rise to 3.2 million (Tang et al., 2019). Primary open-angle glaucoma (POAG) accounts for the majority of glaucoma cases. Because of the high prevalence and occult symptoms of POAG, screening and early detection of POAG are crucial for timely vision preservation and reduction of economic burden (Burr et al., 2007; Tang et al., 2019). However, the early detection of POAG is challenging in community settings because the diagnosis of glaucoma relies on expensive and finely calibrated apparatuses. Existing diagnostic tools include optical coherence tomography (OCT) and standard automated perimetry (SAP). While early structural deficits associated with POAG can be efficiently detected with OCT, SAP testing is still the most prevalent functional method for evaluating vision loss severity and progression. However, the SAP test is subjective, requires the cooperation of the patient, and does not always correlate with POAG retinal structural defects (Quigley et al., 1988; Leung et al., 2011).

The pupillary light response (PLR) relies on the integrity of the connection between the outer (rods, cones) and inner (ipRGC projection) retinal photoreception (Altimus et al., 2010; Chen et al., 2011; Lucas, 2013; Barrionuevo et al., 2023). Retinal photoreception is dependent on the functional integrity of rods, cones, and intrinsically photosensitive retinal ganglion cells (ipRGCs) (Berson et al., 2002; Dacey et al., 2005; Altimus et al., 2010; Lucas, 2013; Kelbsch et al., 2019; Mure et al., 2019; Rukmini et al., 2019; Ahmadi et al., 2020; Aranda and Schmidt, 2021). ipRGCs comprise a small proportion of the total population of RGCs. In addition to receiving inputs from rods and cones, ipRGCs also respond to light (high illuminance and peak sensitivity at ~482 nm) even when isolated from the rest of the retina (Gamlin et al., 2007; Mure et al., 2019; Aranda and Schmidt, 2021; Gamlin, 2022). These ipRGCs project throughout the brain, mediating a wide array of functions, including visual perception and non-image-forming visual functions [PLR, circadian rhythms, sleep, and awakeness (Berson et al., 2002; Chen et al., 2011; Aranda and Schmidt, 2021)]. Although ipRGCs comprise a small portion of the RGC population, ipRGC malfunction can be detected in POAG patients (Gracitelli et al., 2015; Rukmini et al., 2015; Obara et al., 2016; Arevalo-Lopez et al., 2023). The contribution of rods, cones, and ipRGCs to the overall activity that drives the PLR can be biased using certain light intensities, wavelengths, and durations (Kardon et al., 2009; Park et al., 2011; Kelbsch et al., 2019; Barrionuevo et al., 2023).

Inter-eye asymmetry between direct and consensual PLR, or the relative afferent pupillary defect (RAPD), is an informative clinical sign indicating unilaterally or asymmetrically affected eyes (Schiefer et al., 2012; Chang et al., 2013). Glaucoma damage is often more severe in one eye than in the other because of asymmetric loss of retinal nerve fibers (Wang et al., 2004; Broman et al., 2008; Chang et al., 2013). RAPD is often clinically detectable using the swinging flashlight test (SFT) in patients with POAG, correlating with retinal nerve fiber layer (RNFL) loss (Chang et al., 2013; Pillai et al., 2019). However, the SFT is subjective and dependent on the clinician's experience. The objective and quantifiable assessment of RAPD should be performed using binocular pupillometry devices. Thus, chromatic pupillometry could be used as a tool to evaluate the integrity of morphological and functional defects in various diseases, including POAG (Schiefer et al., 2012; Chang et al., 2013; Kelbsch et al., 2016; Meneguette et al., 2019; Pillai et al., 2019). The chromatic light stimulus in pupillometry in previous studies often utilized the Ganzfeld bowl (Kardon et al., 2009; Gracitelli et al., 2014; Rukmini et al., 2015; Kelbsch et al., 2016; Najjar et al., 2018) or Maxwellian view (Feigl et al., 2011; Adhikari et al., 2016). Despite the high effectiveness of chromatic pupillometry in detecting glaucoma, most commercially available pupillometry devices are clunky, expensive, and unsuitable for routine clinical use.

This study aimed to use integrated infrared video pupillography (IVP) with binocular occulted head mount displays (HMDs) and ramping-up lighting stimuli designed with sequential tests, to lower the environmental requirements of the examination. We applied the full-field stimuli to evaluate the global response of rod-, cone-, and ipRGC-weighted PLR sequentially and the intermittent chromatic flashes to evaluate the inter-eye asymmetry of PLR. We extracted the PLR features to correlate these functions with different severities of glaucoma. Furthermore, we evaluated the performance of our binocular chromatic pupillometer as a potential diagnostic tool for evaluating POAG. To our knowledge, this is the first investigation that employs alternating chromatic stimuli for RAPD testing in pupillometry studies of POAG patients.

## 2. Materials and methods

### 2.1. Participants

Seventy-four patients with POAG (26 women and 48 men) and 23 visually healthy controls (12 women and 11 men) were included in this clinic-based, cross-sectional study conducted between May 2021 and October 2022. Based on previous studies (Feigl et al., 2011; Adhikari et al., 2016), a sample size calculation determined that 23 cases in each group were required to achieve 85% power for detecting a significant mean difference of 9.0% in the post-illumination pupillary response between glaucomatous eyes and healthy eyes.

Glaucoma participants were recruited from the Eye and ENT Hospital of Fudan University (Shanghai, China). The research followed the tenets of the Declaration of Helsinki, and the study was approved by the human subjects review committee of the Eye and ENT Hospital of Fudan University. Written informed consent was obtained from each participant. All participants underwent comprehensive ophthalmologic examinations including best-corrected visual acuity, intraocular pressure measurement, slit-lamp stereo biomicroscopy, color vision (Ishihara plates, Kanehara & Co, Tokyo, Japan), and direct ophthalmoscopy, OCT, and SAP. Patients with clinically confirmed POAG were identified by a fellowship-trained specialist as having glaucomatous optic neuropathy (loss of the neuroretinal rim with a vertical cup–disk ratio of >0.7 and/or notching with a nerve fiber layer defect due to glaucoma) and a compatible visual field defect (Najjar et al., 2021). Patients were divided into the mild–moderate stage [mean deviation (MD) scores < 12 dB] and severe stage (MD scores ≥ 12 dB) groups according to Hodapp–Parish–Anderson's criteria. The healthy controls had no ocular or systemic pathology, no corneal opacity, lenticular opacification < grade 2 (LOCS III), and no history of uveitis. Participants were excluded if they had retinopathies, other causes of optic neuropathy or ocular motor disorders; were taking psychotropics or other medications that could affect the PLR; or had pupillary abnormalities other than RAPD. Participants who were diagnosed with neurologic disorders were also excluded.

### 2.2. SAP tests

SAP was evaluated using the Octopus 900 perimeter (Haag-Streit, Koeniz, Switzerland) and the G standard white/white TOP program. All SAP tests were required to meet reliability criteria. SAP tests were defined as abnormal when they met one of the following conditions: (1) the presence of three or more adjacent points in the superior or inferior field with *p* < 5% probability of normal range and one or more points with *p* < 1% probability of normal range or (2) the presence of two or more adjacent points with *p* < 1% probability of normal range and one or more points with *p* < 2% probability (Wen et al., 2015). POAG patients were divided into the mild–moderate stage [mean deviation (MD) scores < 12 dB] and severe stage (MD scores ≥ 12 dB) groups according to Hodapp–Parish–Anderson's criteria.

### 2.3. OCT examinations

Optical coherence tomography was performed with the RTVue-100 spectral domain optical coherence tomography (OCT; Optovue, Inc., Fremont, CA, USA), and the standard glaucoma protocol was applied, including a three-dimensional optic disk scan for the definition of the disk margin, an optic nerve head (ONH) scan, and a standard ganglion cell complex (GCC) scan.

### 2.4. Pupillometer apparatus and calibration

The setup consisted of an HTC VIVE Pro Eye, with an integrated eye tracker (Tobii Pro, Tobii Technology, Stockholm, Sweden). The visual stimuli were generated in Unity 2018, transmitted to the HMD utilizing SteamVR 2.0, and run on a laptop computer (MEHREV Z2, NVIDIA GeForce GTX 2060 i7-10870H @2.20 GHz, 16 GB RAM). Participants were fitted with HMDs that used near-infrared (940 nm) illuminating diodes and infrared cameras to track pupil data (sample rate 90 Hz). The pupil diameter of the eye was obtained through SRanipal SDK supported by Tobii XR and was calculated by multiplying the diameter of the pupil on the acquired image and by a scaling distance factor between the eye and the sensor. The stimuli were presented to one eye, and the pupillary data from both eyes were recorded. Both eyes were tested in turn. The stimuli consisted of short-wavelength (“blue” dominant wavelength of 465 nm) and long-wavelength (“red” dominant wavelength of 642 nm) pulses of light according to different experiments. Stimulus wavelength and luminance were verified using a spectroradiometer (Konica CS-100A; Konica Minolta, Inc., Tokyo, Japan).

### 2.5. Stimuli and procedures of PLR test

**Figure 1:**
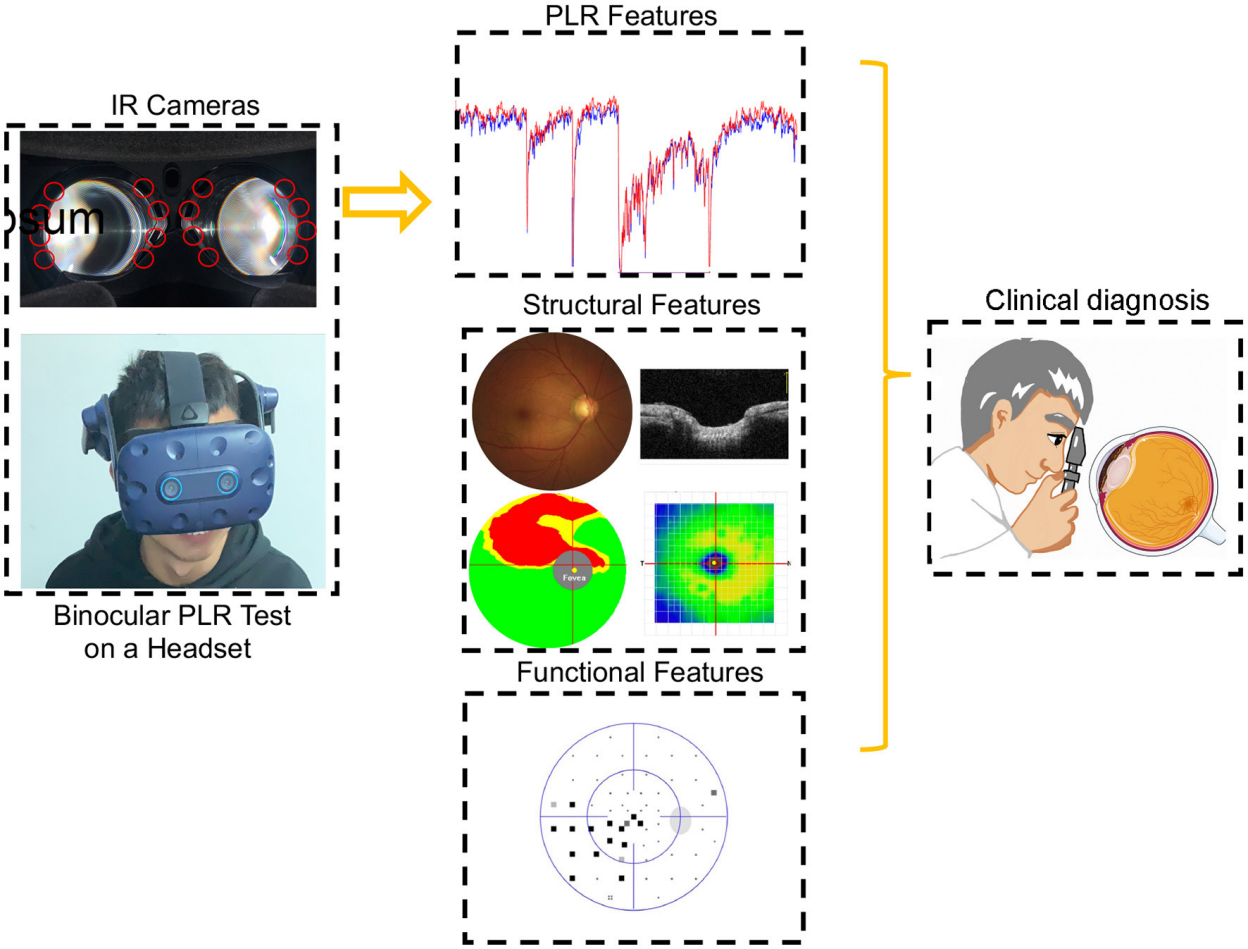
Participants during the PLR test. The binocular PLR test is performed using a headset. The entire testing program could be carried out at one time in a room with normal illumination. The diagnostic accuracy of PLR features was evaluated after comprehensive ophthalmologic examinations.

**Figure 2:**
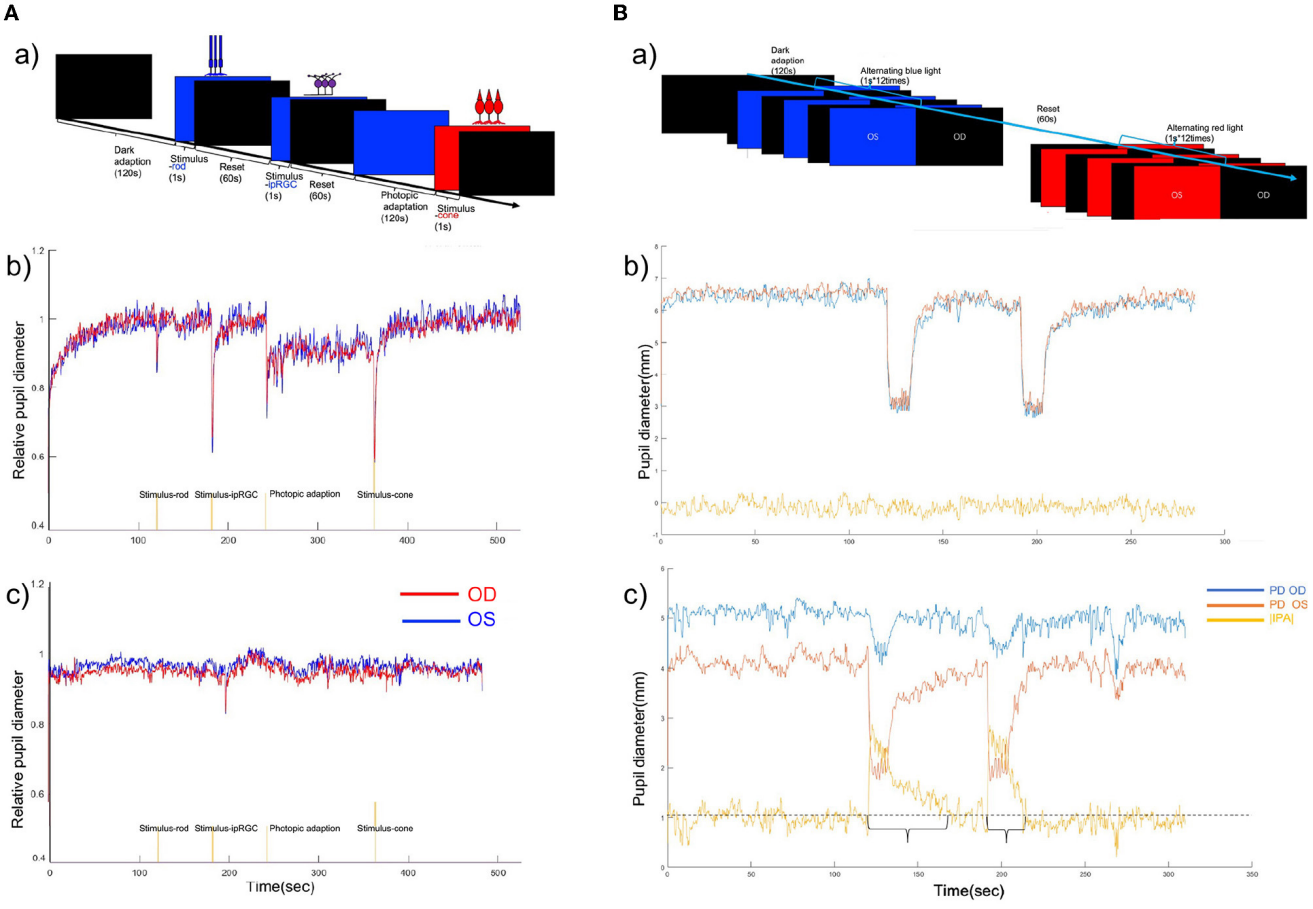
**(A)** (a) Chromatic pupillometry protocol of full-field stimuli. (b) Binocular PLR curve of a healthy participant under full-field stimuli. **(c)** Binocular PLR curve of a participant with POAG. A reduced response was seen to the rod-, cone-, and ipRGC-weighted stimuli. The red line shows the relative pupil diameter curve of the right eye, and the blue line shows the relative pupil diameter curve of the left eye. **(B)** (a) Timeline for intermittent blue and red light flash-induced RAPD. (b) Binocular PLR and infrared pupillary asymmetry (IPA) curve of a healthy participant during intermittent blue and red light flashes. IPA values during the alternating blue and red light flashes. The red line shows the pupil diameter of the left eye, the blue line shows the relative pupil diameter of the right eye, and the yellow line shows the IPA value. The IPA value was calculated as follows: |IPA| = |pupil size (left)-pupil size (right)|. (c) Binocular PLR and IPA curve of a patient with POAG during Experiment 2. The IPA value was >1 mm during the intermittent blue and red light flashes in this case.

Experiment 1: PLR was induced by full-field chromatic light stimuli designed to activate the rod-, cone-, and melanopsin-weighted PLRs (Park et al., 2011). Both eyes were tested in turn, while the direct and consensual PLR was recorded by the binocular IVP (Figures 1, 2A). After calibration of the HMDs, dark adaption was performed by displaying a dark screen for 120 s. For the rod-weighted PLR test, a blue flash with a luminance of 0.1 cd/m^2^ was presented. For the melanopsin-dependent PLR test, a blue flash with a high luminance of 100 cd/m^2^ was shown. For the cone-weighted PLR tests, the tested eye was first presented with a 6-cd/m^2^ blue field for 2 min to suppress rod activity, followed by a red flash (10 cd/m^2^).

Experiment 2: A quantitative assessment of chromatic light-induced RAPD was designed to assess the inter-eye asymmetry of glaucomatous impairment of the anterior afferent visual pathways. The program alternatively applied blue (100 cd/m^2^) or red (100 cd/m^2^) flash to both eyes (1 s^*^12 times). The direct and consensual response was recorded (Figure 2B).

### 2.6. PLR data acquisition and feature extraction

During the experiment, the real-time pupil diameter was recorded (90 Hz). Data were analyzed offline using custom scripts programmed in MATLAB (MathWorks, Inc., Natick, MA, USA), which automated interpolated eye movement and filtered short-time (<500-ms) eye blinks.

In Experiment 1, the following components were extracted:

1. Baseline pupil diameter (BPD; mm): mean pupillary diameter during the initial 10 s before light onset.
2. Rod-weighted PLR: maximum constriction amplitude induced by rod-weighted PLR (amplitude rod; percent in %) = BPD-maximum constriction pupil diameter/BPD^*^100%.
3. Time to maximal constriction induced by rod-weighted PLR (T rod constriction; s): the interval between the beginning of the rod-weighted stimulus and peak constriction.
4. Time to dilation induced by rod-weighted PLR (T rod dilation; s): the interval between the beginning of the rod-weighted stimulus and the recovery of the pupil diameter to 90% BPD.
5. Constriction velocity of rod-weighted PLR (Vel rod constriction; mm/s): velocity of pupillary constriction induced by rod-weighted stimulus.
6. Dilation velocity of rod-weighted PLR (Vel rod dilation; mm/s): velocity of pupillary dilation to 90% BPD following rod-weighted stimulus.
7. ipRGC-weighted PLR: In addition to the parameters (amplitude, latency, and velocity) measured in the rod-weighted PLR test, the post-illumination pupillary response (PIPR) was also calculated. Sustained response was defined as the pupil diameter at 6 s after melanopsin-weighted stimulus flash offset. PIPR: BPD-PD _at6safterflashoffset_/BPD^*^100%.
8. Cone-weighted PLR: The constriction amplitude, latency, velocity of constriction, and dilation were calculated as above mentioned.

In Experiment 2, the infrared pupillary asymmetry (IPA) curve was recorded during alternating stimuli (Meneguette et al., 2019).

IPA was recorded as a positive value as follows: |IPA| (blue/red flash induced) = |pupil diameter (OD)– pupil diameter (OS)|. The duration of an IPA >1 mm was calculated (intermittent blue/red flash induced).

### 2.7. Statistical analysis

Ophthalmologic examination outcomes and pupillometric features are represented as the means ± standard deviation or median (interquartile range). Comparisons between controls and the different glaucoma severity groups were evaluated using a one-way analysis of variance (ANOVA) or Kruskal–Wallis one-way analysis of variance on ranks test, and a *post-hoc* analysis (LSD) or Dunn's method was further performed to identify significant group differences. Comparisons between the two groups were evaluated using the one-way ANOVA or the Wilcoxon rank-sum test (Table 1). The correlation between pupillometric features and clinical measures (e.g., mean deviation and RNFL thickness) was assessed using Pearson's correlation analysis or Spearman's ranks correlation coefficient. The correlation between the combined effect of multiple pupillometric features and ophthalmologic examination outcomes was assessed using multiple linear regression. Receiver operating characteristic (ROC) curve analysis was performed, and the area under the ROC curve (AUC) was calculated to compare the diagnostic value using the pROC package and reportROC package. The sensitivity, specificity, and 95% CI were calculated at the best statistical cutoff (highest Youden's *J*). The relationship between the cumulative effect of pupillometric features and glaucoma severity was assessed using logistic regression. Comparison between the AUCs of pupillometric features, OCT, or SAP was performed using DeLong's method with Bonferroni correction. The threshold for significance for all statistical tests was set at 0.05. Statistical procedures were performed using R Version.4.2.2: A Language and Environment for Statistical Computing (R Core Team, Vienna, Austria).

**Table 1:** Ocular characteristics and pupillometric parameters in full-field stimuli of the study eyes in control and POAG.

	**Healthy control**	**Glaucoma**	**Mild–moderate glaucoma**	**Severe glaucoma**	**P-value**			
					**Control vs. glaucoma**	**Control vs. mild–moderate glaucoma**	**Control vs. severe glaucoma**	**Mild–moderate glaucoma vs. severe glaucoma**
***N***	46	148	94	54	NA	NA	NA	NA
Age (mean ± SD; years)	42.00 (11.08)	42.46 (13.81)	40.67 (12.34)	45.15 (15.64)	NA	NA	NA	NA
MD, median (IQR; dB)	2.45 (1.40, 3.08)	7.24 (3.83, 15.80)	4.57 (2.59, 6.95)	18.81 (15.50, 25.45)	**< 0.001**	**< 0.001**	**< 0.001**	**< 0.001**
RNFL, median (IQR; μm)	99.00 (91.00, 103.00)	73.00 (61.00, 86.00)	80.00 (72.00, 92.00)	59.00 (52.25, 67.00)	**< 0.001**	**< 0.001**	**< 0.001**	**< 0.001**
C/D ratio, median (IQR)	0.44 (0.29, 0.55)	0.86 (0.76, 0.92)	0.82 (0.69, 0.89)	0.92 (0.86, 0.95)	**< 0.001**	**< 0.001**	**< 0.001**	**< 0.001**
Rim area, median (IQR; mm^2^)	1.25 (1.04, 1.39)	0.66 (0.46, 0.98)	0.80 (0.57, 1.07)	0.46 (0.32, 0.70)	**< 0.001**	**< 0.001**	**< 0.001**	**< 0.001**
Cup volume, median (IQR; mm^3^)	0.05 (0.01, 0.22)	0.45 (0.25, 0.74)	0.41 (0.20, 0.66)	0.56 (0.36, 0.86)	**< 0.001**	**< 0.001**	**< 0.001**	**0.006**
GCC, median (IQR; μm)	95.00 (92.25, 97.75)	78.00 (61.75, 88.00)	83.00 (78.00, 92.00)	60.00 (57.00, 67.75)	**< 0.001**	**< 0.001**	**< 0.001**	**< 0.001**
BPD, median (IQR; mm)	6.03 (5.45, 6.51)	5.67 (4.97, 6.43)	5.90 (5.16, 6.43)	5.44 (4.81, 6.33)	0.060	0.272	**0.026**	0.107
Amplitude rod, median (IQR)	0.25 (0.20, 0.29)	0.18 (0.13, 0.25)	0.19 (0.15, 0.26)	0.15 (0.09, 0.24)	**0.001**	**0.032**	**< 0.001**	**0.032**
T rod constriction, median (IQR; s)	1.11 (0.95, 1.27)	1.12 (0.93, 1.39)	1.04 (0.90, 1.29)	1.27 (0.98, 1.52)	0.531	0.546	**0.033**	**0.002**
Vel rod constriction, median (IQR; mm/s)	1.31 (1.00, 1.59)	0.99 (0.63, 1.30)	1.08 (0.78, 1.42)	0.65 (0.42, 1.18)	**0.001**	**0.031**	**< 0.001**	**0.004**
T rod dilation, median (IQR; s)	14.71 (8.42, 17.34)	16.92 (14.09, 17.77)	16.00 (12.90, 17.60)	17.46 (16.13, 17.97)	**0.010**	0.196	**< 0.001**	**0.003**
Vel rod dilation, median (IQR; mm/s)	0.14 (0.11, 0.33)	0.12 (0.05, 0.33)	0.14 (0.09, 0.32)	0.10 (0.05, 0.41)	0.143	0.562	**0.04**	**0.043**
Amplitude ipRGC, median (IQR)	0.45 (0.39, 0.49)	0.37 (0.32, 0.44)	0.38 (0.34, 0.45)	0.34 (0.24, 0.40)	**< 0.001**	**0.004**	**< 0.001**	**0.004**
T ipRGC constriction, median (IQR; s)	1.46 (1.38, 1.53)	1.45 (1.33, 1.58)	1.44 (1.36, 1.53)	1.48 (1.31, 1.64)	0.865	NA	NA	NA
Vel ipRGC constriction, median (IQR; mm/s)	1.74 (1.57, 1.89)	1.57 (1.13, 1.88)	1.64 (1.30, 1.93)	1.48 (0.94, 1.68)	**0.005**	0.126	**< 0.001**	**0.003**
T ipRGC dilation, median (IQR; s)	15.64 (12.48, 16.68)	17.42 (16.89, 18.06)	17.31 (16.67, 17.63)	17.86 (17.23, 18.69)	**< 0.001**	**< 0.001**	**< 0.001**	**0.001**
Vel ipRGC dilation, median (IQR; mm/s)	0.16 (0.15, 0.20)	0.15 (0.11, 0.19)	0.16 (0.13, 0.20)	0.12 (0.08, 0.16)	**0.009**	0.233	**< 0.001**	**0.001**
Sustained response, median (IQR; mm)	5.43 (4.85, 6.11)	4.97 (4.09, 5.65)	5.09 (4.52, 5.96)	4.50 (4.05, 5.29)	**0.002**	0.051	**< 0.001**	**0.006**
PIPR, median (IQR)	0.09 (0.06, 0.11)	0.13 (0.07, 0.17)	0.11 (0.06, 0.15)	0.15 (0.11, 0.19)	**< 0.001**	**0.037**	**< 0.001**	**0.001**
Amplitude cone, median (IQR)	0.49 (0.45, 0.52)	0.41 (0.35, 0.47)	0.43 (0.38, 0.47)	0.37 (0.27, 0.44)	**< 0.001**	**0.001**	**< 0.001**	**< 0.001**
T cone constriction, median (IQR; s)	1.33 (1.25, 1.41)	1.39 (1.29, 1.46)	1.40 (1.30, 1.46)	1.39 (1.24, 1.48)	**0.037**	NA	NA	NA
Vel cone constriction, median (IQR; mm/s)	3.73 (3.36, 3.96)	3.37 (2.63, 3.90)	3.54 (3.00, 4.00)	2.87 (2.32, 3.55)	**0.014**	0.269	**< 0.001**	**0.001**
T cone dilation, median (IQR; s)	17.37 (16.16, 17.68)	17.50 (14.96, 17.69)	17.46 (13.84, 17.66)	17.54 (16.60, 17.76)	0.957	NA	NA	NA
Vel cone dilation, median (IQR; mm/s)	0.17 (0.16, 0.20)	0.16 (0.11, 0.19)	0.18 (0.15, 0.22)	0.11 (0.08, 0.16)	0.055	0.853	**< 0.001**	**< 0.001**

A Kruskal–Wallis one-way ANOVA on ranks was used to compare controls, mild–moderate stage, and severe stage glaucoma groups. A post-hoc pairwise comparison between the three groups was done using Dunn's method. Comparison between the two groups was evaluated using the Wilcoxon rank-sum test.

IQR, interquartile range; MD, mean deviation; RNFL, retinal nerve fiber layer; GCC, ganglion cell complex; BPD, baseline pupil diameter; t, time; vel, velocity; ipRGC, intrinsically photosensitive retinal ganglion cell; PIPR, post-illumination pupil response.

Bold value means statistically significant.

## 3. Results

### 3.1. Participant characteristics

This study included 194 eyes from 97 participants, including 148 eyes from 74 patients with POAG, and 46 eyes from 23 healthy controls. The age in the control group was 42.00 ± 11.08 years, and the age in the glaucoma group was 42.46 ±13.81 years. POAG patients were further divided into mild–moderate and severe glaucoma groups. The description of the clinical characteristics and the pupillometric parameters under full-field stimuli of all the groups are provided in Table 1.

#### 3.1.1. Pupillometric parameters in the full-field stimuli experiment

In the full-field stimuli experiments, the relative pupillary diameter curves were significantly reduced at all light intensities for both red and blue lights in the glaucoma group compared with the control group (Figure 2A, Table 1). Multiple PLR features were different between the groups. In the full-field stimuli experiments, the amplitude of transient constriction in response to different light stimuli was significantly different between the control and the POAG group (*p*_Amplituderod_ = 0.001; *p*_AmplitudeipRGC_ < 0.001; *p*_Amplitudecone_ < 0.001). The velocity of pupil constriction was slower in POAG patients (*p* < 0.001 in three comparisons). In rod-weighted PLR and ipRGC-weighted PLR, dilation after transient constriction was prolonged in POAG patients (*p*_Troddilation_ = 0.01; *p*_TipRGCdilation_ < 0.001), and no obvious delayed dilation was observed in cone-weighted PLR (*p* = 0.143). However, the velocity of dilation was significantly slower in patients with POAG who responded to the three stimuli (*p*_Velroddilation_ = 0.024; *p*_VelipRGCdilation_ < 0.001; *p*_Velconedilation_ < 0.001). The post-illumination response of ipRGC-weighted PLR was different between healthy controls and patients with different severities of POAG (Table 1).

#### 3.1.2. Diagnostic value of pupillometric parameters in full-field stimuli experiments

**Figure 3:**
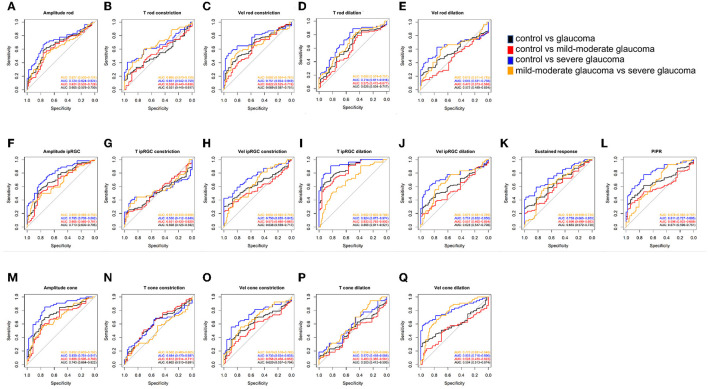
ROC curves showing the diagnostic values of PLR features in Experiment 1. **(A–E)** Rod-weighted PLR; **(F–L)** ipRGC-weighted PLR; **(M–Q)** cone-weighted PLR. Black line = AUC for discriminating control vs. glaucoma, red line = AUC for discriminating control vs. mild–moderate glaucoma; blue line = AUC for discriminating control vs. severe glaucoma; orange line= AUC for discriminating mild–moderate glaucoma vs. severe glaucoma. Data are represented as average (95% CI). ROC, receiver operating characteristic; PLR, pupil light response; ipRGC, intrinsically photosensitive retinal ganglion cell; AUC, area under the ROC curve; T, time; Vel, velocity; PIPR, post-illumination pupil response.

The diagnostic value of the pupillometric parameters in full-field stimuli experiments is provided in Table 2 and Figure 3. The variable with the highest AUC was time to max dilation in ipRGC-weighted PLR (T ipRGC dilation; AUC = 0.866, sensitivity = 0.662, specificity = 0.913). This parameter also presented a good AUC for diagnosing mild–moderate stage POAG (AUC = 0.833, sensitivity = 0.585, specificity = 0.913). The second highest AUC was the constriction amplitude response to cone-weighted stimuli (amplitude cone; AUC = 0.743, sensitivity = 0.696, specificity = 0.739), followed by the constriction amplitude response to ipRGC-weighted stimuli (amplitude ipRGC; AUC = 0.713, sensitivity = 0.541, specificity = 0.826) and PIPR (AUC = 0.674, sensitivity = 0.615, specificity = 0.739). The highest AUC for diagnosing the progression of POAG was the velocity of dilation in cone-weighted PLR (Vel cone dilation; AUC = 0.770, sensitivity = 0.667, specificity = 0.766).

**Table 2:** Diagnostic value of pupillometric parameters in full-field stimuli.

	**Control vs. glaucoma**			**Control vs. mild–moderate glaucoma**			**Control vs. severe glaucoma**			**Mild–moderate glaucoma vs. severe glaucoma**		
	**AUC**	**Sensitivity**	**Specificity**	**AUC**	**Sensitivity**	**Specificity**	**AUC**	**Sensitivity**	**Specificity**	**AUC**	**Sensitivity**	**Specificity**
BPD	0.592 (0.505, 0.679)	0.514 (0.433, 0.594)	0.674 (0.538, 0.809)	0.554 (0.457, 0.652)	0.181 (0.103, 0.259)	0.978 (0.936, 1.000)	0.658 (0.551, 0.765)	0.611 (0.481, 0.741)	0.696 (0.563, 0.829)	0.593 (0.497, 0.688)	0.537 (0.404, 0.670)	0.681 (0.587, 0.775)
Amplitude rod	0.665 (0.579, 0.750)	0.588 (0.509, 0.667)	0.761 (0.638, 0.884)	0.631 (0.533, 0.728)	0.543 (0.442, 0.643)	0.761 (0.638, 0.884)	0.724 (0.624, 0.824)	0.667 (0.541, 0.792)	0.761 (0.638, 0.884)	0.627 (0.530, 0.724)	0.537 (0.404, 0.670)	0.713 (0.621, 0.804)
T rod constriction	0.531 (0.445, 0.617)	0.230 (0.162, 0.298)	0.957 (0.898, 1.000)	0.538 (0.440, 0.636)	0.255 (0.167, 0.343)	0.891 (0.801, 0.981)	0.651 (0.542, 0.759)	0.407 (0.276, 0.538)	0.957 (0.898, 1.000)	0.665 (0.570, 0.759)	0.611 (0.481, 0.741)	0.702 (0.610, 0.795)
Vel rod constriction	0.669 (0.587, 0.751)	0.486 (0.406, 0.567)	0.804 (0.690, 0.919)	0.622 (0.525, 0.719)	0.702 (0.610, 0.795)	0.543 (0.400, 0.687)	0.751 (0.654, 0.849)	0.537 (0.404, 0.670)	0.935 (0.863, 1.000)	0.662 (0.564, 0.761)	0.537 (0.404, 0.670)	0.819 (0.741, 0.897)
T rod dilation	0.626 (0.534, 0.717)	0.804 (0.740, 0.868)	0.457 (0.313, 0.600)	0.575 (0.472, 0.677)	0.787 (0.704, 0.870)	0.435 (0.292, 0.578)	0.714 (0.611, 0.816)	0.889 (0.805, 0.973)	0.500 (0.356, 0.644)	0.666 (0.574, 0.757)	0.741 (0.624, 0.858)	0.553 (0.453, 0.654)
Vel rod dilation	0.572 (0.489, 0.654)	0.324 (0.249, 0.400)	0.891 (0.801, 0.981)	0.530 (0.432, 0.627)	0.160 (0.086, 0.234)	1.000 (1.000, 1.000)	0.645 (0.531, 0.758)	0.630 (0.501, 0.758)	0.761 (0.638, 0.884)	0.613 (0.511, 0.715)	0.630 (0.501, 0.758)	0.681 (0.587, 0.775)
Amplitude ipRGC	0.713 (0.630, 0.795)	0.541 (0.460, 0.621)	0.826 (0.717, 0.936)	0.665 (0.569, 0.761)	0.489 (0.388, 0.590)	0.826 (0.717, 0.936)	0.795 (0.708, 0.882)	0.741 (0.624, 0.858)	0.717 (0.587, 0.848)	0.659 (0.565, 0.753)	0.370 (0.242, 0.499)	0.926 (0.872, 0.979)
T ipRGC constriction	0.508 (0.425, 0.592)	0.236 (0.168, 0.305)	0.957 (0.898, 1.000)	0.531 (0.433, 0.629)	0.191 (0.112, 0.271)	0.978 (0.936, 1.000)	0.530 (0.412, 0.649)	0.389 (0.259, 0.519)	0.913 (0.832, 0.994)	0.551 (0.443, 0.658)	0.352 (0.224, 0.479)	0.915 (0.858, 0.971)
Vel ipRGC constriction	0.638 (0.559, 0.717)	0.304 (0.230, 0.378)	1.000 (1.000, 1.000)	0.573 (0.480, 0.667)	0.372 (0.275, 0.470)	0.870 (0.772, 0.967)	0.750 (0.655, 0.845)	0.426 (0.294, 0.558)	1.000 (1.000, 1.000)	0.654 (0.562, 0.745)	0.796 (0.689, 0.904)	0.457 (0.357, 0.558)
T ipRGC dilation	0.866 (0.811, 0.921)	0.662 (0.586, 0.738)	0.913 (0.832, 0.994)	0.833 (0.763, 0.902)	0.585 (0.486, 0.685)	0.913 (0.832, 0.994)	0.924 (0.875, 0.974)	0.907 (0.830, 0.985)	0.826 (0.717, 0.936)	0.692 (0.603, 0.780)	0.500 (0.367, 0.633)	0.819 (0.741, 0.897)
Vel ipRGC dilation	0.628 (0.547, 0.708)	0.493 (0.413, 0.574)	0.783 (0.663, 0.902)	0.557 (0.460, 0.654)	0.191 (0.112, 0.271)	0.978 (0.936, 1.000)	0.751 (0.652, 0.850)	0.611 (0.481, 0.741)	0.870 (0.772, 0.967)	0.675 (0.581, 0.769)	0.648 (0.521, 0.776)	0.702 (0.610, 0.795)
Sustained response	0.655 (0.572, 0.738)	0.277 (0.205, 0.349)	1.000 (1.000, 1.000)	0.596 (0.499, 0.693)	0.202 (0.121, 0.283)	1.000 (1.000, 1.000)	0.758 (0.665, 0.850)	0.444 (0.312, 0.577)	0.978 (0.936, 1.000)	0.641 (0.549, 0.734)	0.574 (0.442, 0.706)	0.713 (0.621, 0.804)
PIPR	0.674 (0.598, 0.751)	0.615 (0.536, 0.693)	0.739 (0.612, 0.866)	0.596 (0.503, 0.689)	0.340 (0.245, 0.436)	0.935 (0.863, 1.000)	0.811 (0.727, 0.895)	0.778 (0.667, 0.889)	0.739 (0.612, 0.866)	0.670 (0.582, 0.757)	0.926 (0.856, 0.996)	0.383 (0.285, 0.481)
Amplitude cone	0.743 (0.664, 0.822)	0.696 (0.622, 0.770)	0.739 (0.612, 0.866)	0.688 (0.595, 0.780)	0.574 (0.475, 0.674)	0.783 (0.663, 0.902)	0.839 (0.761, 0.917)	0.852 (0.757, 0.947)	0.739 (0.612, 0.866)	0.692 (0.603, 0.782)	0.556 (0.423, 0.688)	0.777 (0.692, 0.861)
T cone constriction	0.602 (0.513, 0.691)	0.655 (0.579, 0.732)	0.565 (0.422, 0.708)	0.612 (0.514, 0.711)	0.511 (0.410, 0.612)	0.717 (0.587, 0.848)	0.584 (0.470, 0.697)	0.667 (0.541, 0.792)	0.565 (0.422, 0.708)	0.502 (0.400, 0.605)	0.278 (0.158, 0.397)	0.830 (0.754, 0.906)
Vel cone constriction	0.620 (0.537, 0.704)	0.372 (0.294, 0.449)	0.891 (0.801, 0.981)	0.556 (0.458, 0.653)	0.415 (0.315, 0.514)	0.761 (0.638, 0.884)	0.733 (0.634, 0.833)	0.556 (0.423, 0.688)	0.891 (0.801, 0.981)	0.670 (0.578, 0.763)	0.556 (0.423, 0.688)	0.755 (0.668, 0.842)
T cone dilation	0.503 (0.412, 0.593)	0.122 (0.069, 0.174)	1.000 (1.000, 1.000)	0.537 (0.439, 0.635)	0.287 (0.196, 0.379)	0.870 (0.772, 0.967)	0.572 (0.459, 0.685)	0.185 (0.082, 0.289)	1.000 (1.000, 1.000)	0.594 (0.500, 0.688)	0.944 (0.883, 1.000)	0.245 (0.158, 0.332)
Vel cone dilation	0.594 (0.513, 0.674)	0.318 (0.243, 0.393)	0.978 (0.936, 1.000)	0.526 (0.430, 0.623)	0.479 (0.378, 0.580)	0.696 (0.563, 0.829)	0.803 (0.716, 0.890)	0.574 (0.442, 0.706)	0.978 (0.936, 1.000)	0.770 (0.691, 0.848)	0.667 (0.541, 0.792)	0.766 (0.680, 0.852)

Data are represented as average (95% CI).

AUC, area under the ROC curve; BPD, baseline pupil diameter; vel, velocity; ipRGC, intrinsically photosensitive retinal ganglion cell; PIPR, post-illumination pupil response.

#### 3.1.3. Correlation of PLR features with clinical parameters

**Figure 4:**
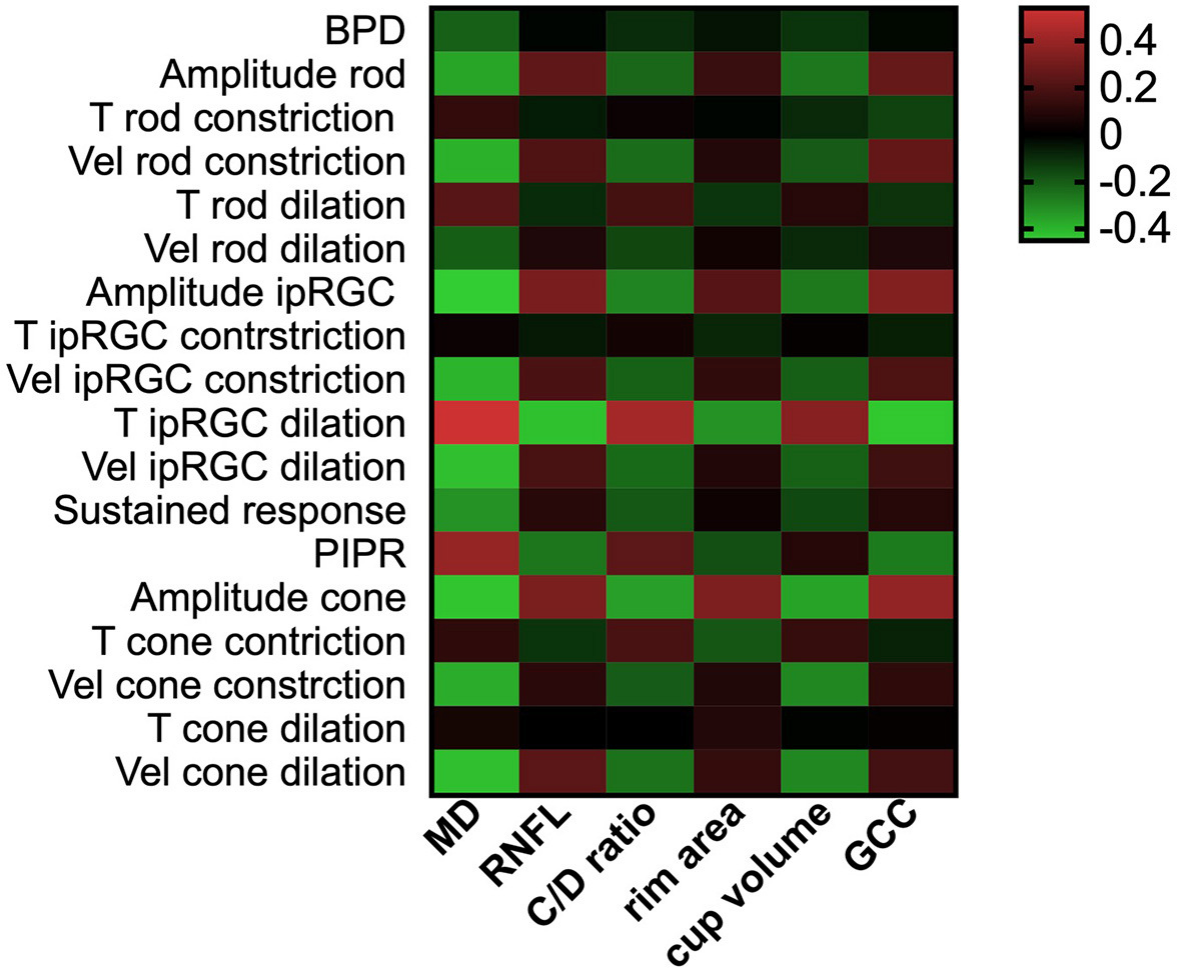
PLR features correlated with retinal structural and functional clinical parameters. The heatmaps show Pearson's correlation coefficient values for retinal structural parameters and visual field median defect vs. PLR features. A greater red color indicates higher positive correlation coefficient values, and a greater green color indicates higher negative coefficient values. PLR, pupil light response; MD, median defect; RNFL, retinal nerve fiber layer; GCC, ganglion cell complex; BPD, baseline pupil diameter; T, time; Vel, velocity; ipRGC, intrinsically photosensitive retinal ganglion cell; PIPR, post-illumination pupil response.

The correlation between PLR features under full-field stimuli and current parameters to assess the severity of POAG is presented in Figure 4. The impaired PLR was associated with greater visual field loss, thinner RNFL thickness, and cupping of the optic disk. Among the PLR to full-field stimuli, the correlation between T ipRGC dilation and current parameters for assessing POAG severity was the strongest (*r*_MD_ = 0.538, *p*_MD_ < 0.001; *r*_RNFL_ = −0.428, *p*_RNFL_ < 0.001; *r*_C/Dratio_ = 0.428, *p*_C/Dratio_ < 0.001; *r*_rimarea_ = −0.324, *p*_rimarea_ < 0.001; *r*_cupvolume_ = 0.350, *p*_cupvolume_ < 0.001; *r*_GCC_ = −0.441, *p*_GCC_ = < 0.001). The constriction amplitude responded to ipRGC-weighted stimuli (*r*_MD_ = −0.451, *p*_MD_ < 0.001; *r*_RNFL_ = 0.314, p_RNFL_ < 0.001; *r*_C/Dratio_ = −0.291, *p*_C/Dratio_ < 0.001; *r*_rimarea_ = 0.225, *p*_rimarea_ = 0.002; *r*_cupvolume_ = −0.268, *p*_cupvolume_ < 0.001; *r*_GCC_ = 0.339, *p*_GCC_ = < 0.001) and the constriction amplitude of cone stimuli (*r*_MD_ = −0.436, *p*_MD_ < 0.001; *r*_RNFL_ = 0.322, *p*_RNFL_ < 0.001; *r*_C/Dratio_ = −0.354, *p*_C/Dratio_ < 0.001; *r*_rimarea_ = 0.323, *p*_rimarea_ < 0.001; *r*_cupvolume_ = −0.359, *p*_cupvolume_ < 0.001; *r*_GCC_ = 0.379, *p*_GCC_ = < 0.001) also showed strong correlation with the clinical parameters.

### 3.2. PLR asymmetry in POAG patients and controls

**Figure 5:**
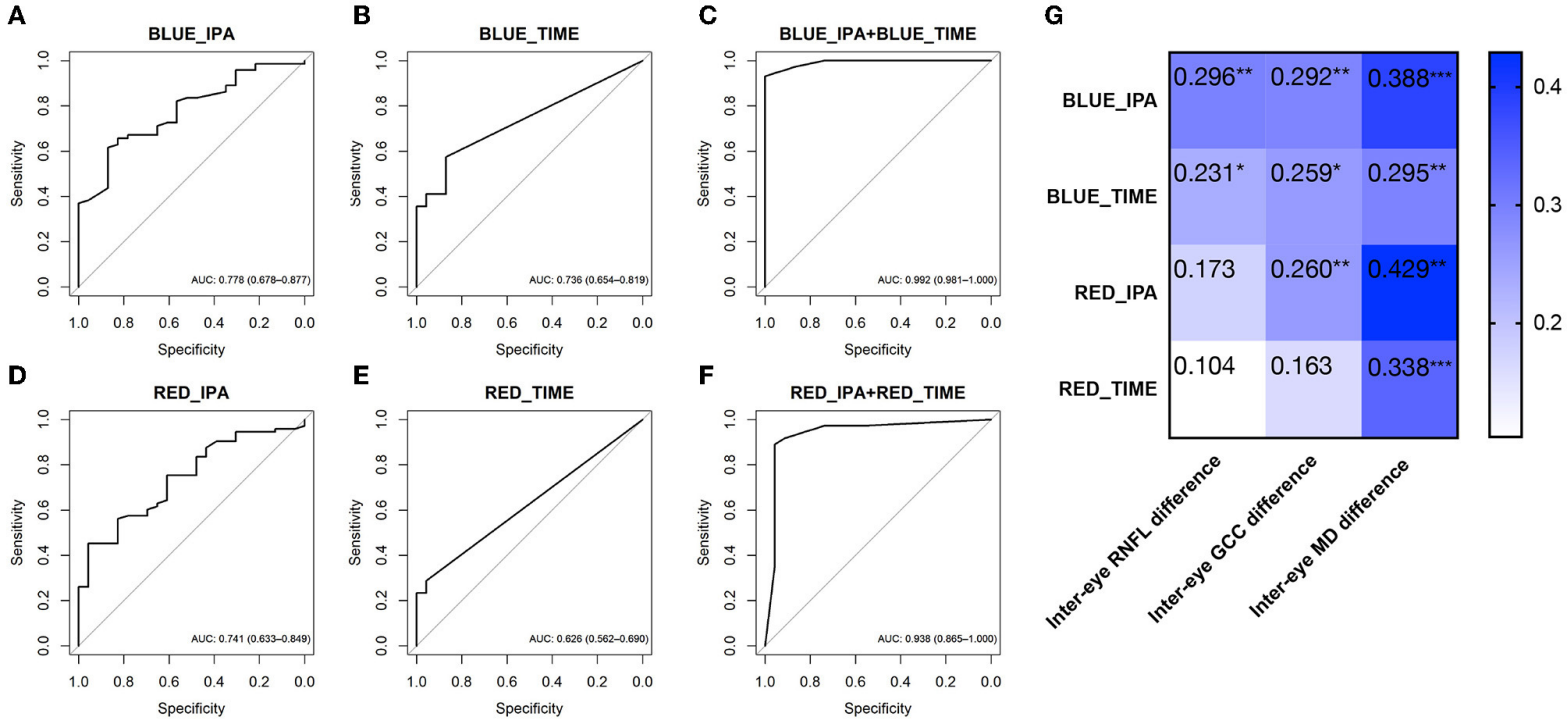
ROC curves showing the diagnostic values of PLR features in Experiment 2 [intermittent blue light flash- **(A–C)** and red light flash-induced RAPD **(D–F)**]. **(G)** The heatmaps show Spearman's rank correlation coefficient values for ocular asymmetric parameters vs. RAPD parameters. The characteristics inside the cells show the rho (*r*_s_) values of Spearman's rank correlation coefficient. *p < 0.05; **p < 0.01; *** *p* < 0.001. Data are represented as average (95% CI). ROC, receiver operating characteristic; AUC, area under the ROC curve; PLR, pupil light response; RAPD, relative afferent pupillary defect; BLUE_IPA, intermittent blue light flash-induced infrared pupillary asymmetry; BLUE_TIME, anisocoria duration during intermittent blue light flashes; RED_IPA, intermittent red light flash-induced infrared pupillary asymmetry; RED_TIME, anisocoria duration during intermittent red light flashes.

In Experiment 2, the IPA induced by intermittent blue or red light flashes was significantly larger in patients with POAG than in healthy controls (Figure 2B and Table 3, *p*_Blue − IPA_ < 0.001, *p*_Red − IPA_ = 0.001). The duration of the anisocoria induced by intermittent blue or red light flashes was significantly longer in patients with POAG than in healthy controls (Figure 2B, Table 3, *p*_Blue − time_ < 0.001, *p*_Red − IPA_ = 0.014). Among 74 POAG patients, 39 (52.7%) presented RAPD during intermittent blue light flashes, and 20 (27.0%) presented RAPD during intermittent red light flashes, which were more frequent than among healthy controls (13.0 and 4.3%, respectively). IPA and anisocoria duration had good diagnostic values (Figures 5A–F; AUC_Blue − IPA_ = 0.778, sensitivity _Blue − IPA_ = 0.616, specificity _Blue − IPA_ = 0.870; AUC_Blue − time_ = 0.736, sensitivity_Blue − time_ = 0.575, specificity _Blue − time_ = 0.870; AUC_Red − IPA_ = 0.741, sensitivity_Red − IPA_ = 0.452, specificity = 0.957; AUC_Red − time_ = 0.626, sensitivity _Red − time_ = 0.288, specificity_Red − time_ = 0.957). The cumulative effect of two paired parameters showed a higher AUC for diagnosing patients with POAG (AUC_Blue − IPA+Blue − time_ = 0.992; AUC_Red − IPA+Red − time_ = 0.992). The deficit in the RAPD tests was correlated with the inter-eye asymmetry of retinal structure and MD score (Figure 5G). In particular, there was a correlation between IPA and inter-eye MD difference (r_Blue − IPA&inter − eyeMDdifference_ = 0.388, *p* < 0.001; *r*_Red − IPA&inter − eyeMDdifference_ = 0.338, *p* < 0.001).

**Table 3:** Pupillary asymmetry characteristics and ocular inter-eye differences in the control and glaucoma patients.

	**Control**	**Glaucoma**	**P-value**
Inter-eye RNFL difference (μm)	3.00 (2.00, 6.50)	9.00 (4.00, 15.00)	< 0.001
Inter-eye GCC difference (μm)	1.00 (0.50, 2.50)	8.00 (3.00, 15.00)	< 0.001
Inter-eye MD difference (dB)	0.60 (0.40, 0.95)	4.20 (1.90, 9.20)	< 0.001
Blue-IPA (mm)	0.63 (0.50, 0.90)	1.03 (0.68, 1.37)	< 0.001
Blue-time (s)	0.00 (0.00, 0.00)	3.80 (0.00, 10.01)	< 0.001
Red-IPA (mm)	0.57 (0.43, 0.70)	0.76 (0.58, 1.11)	0.001
Red-time (s)	0.00 (0.00, 0.00)	0.00 (0.00, 3.80)	0.014

Data presented as mean (interquartile range).

Statistical comparisons between groups were performed using a one-way analysis of variance (ANOVA). RNFL, retinal nerve fiber layer; GCC, ganglion cell complex; MD, mean deviation; IPA, infrared pupillary asymmetry.

## 4. Discussion

In this study, we demonstrated that POAG is associated with altered PLR features induced by chromatic light stimuli using a binocular pupillometer. In full-field stimuli-induced PLR tests, the variable with the highest diagnostic value was time to max dilation in ipRGC-weighted stimuli-induced PLR (T ipRGC dilation), followed by constriction amplitude responding to cone-weighted stimuli (amplitude cone) and constriction amplitude responding to ipRGC-weighted stimuli (amplitude ipRGC) and PIPR. We also highlight a close relationship between time to pupillary redilation to bright blue light with changes in retinal structure (i.e., RNFL thinning) and visual function (MD score). In RAPD tests, the inter-eye PLR asymmetry in patients with POAG was quantified by the IPA value and anisocoria duration, which correlated with the inter-eye asymmetry of retinal structure and MD score. These findings suggest that the impairment of pupillary responses could be detected using binocular pupillometry, and the PLR tests could potentially be used to estimate the severity of POAG and the inter-eye asymmetry in glaucoma severity.

A PLR test using bright white light flashes is commonly used in clinical practice to roughly assess retinal sensitivity. However, a white light flash covers a wide spectrum of wavelengths and does not selectively favor the activation of excitatory photoreceptors (Kardon et al., 2009; Lucas, 2013). Several studies have reported protocols for separating rod, cone, and ipRGC function by setting the irradiance, wavelength, size, and duration of the stimuli (Kardon et al., 2009; Kelbsch et al., 2019; Rukmini et al., 2019). A low-intensity short-wavelength light stimulus primarily stimulates the rod function, and a high-intensity long-wavelength light stimulus evokes the cone activity, whereas a pupil response to high-intensity short-wavelength light stimulus most likely involves a combination of cone and melanopsin activation (Young and Kimura, 2008; Kardon et al., 2009; Chen et al., 2011; Kelbsch et al., 2019). By comparing several stimulus protocols, Lisowska et al. (2017) proposed that for rod-favoring conditions, the stimulus should have a low intensity, and short wavelength (e.g., 4 ms; 0.01 Lux) after prolonged dark adaptation, while for cone-favoring conditions, the stimulus can be brighter, and have a long wavelength (e.g., 1,000 ms; 28 Lux) after 10 min of light adaptation (Kelbsch et al., 2019). The peak-to-trough amplitude of the flicker (0.5 Hz) pupil response to blue test stimuli (with high melanopsin excitation) and red test stimuli (with low melanopsin excitation) can be analyzed to determine the level of interaction between the outer retinal photoreceptors and inner retinal melanopsin (Feigl and Zele, 2014; Joyce et al., 2015; Maynard et al., 2015). In most ipRGC function studies, the PIPR has been assessed using stimuli with a wavelength near the melanopsin optimal sensitivity (~482 nm), and durations ranging from 4 ms to 30 s (Feigl et al., 2011; Park et al., 2011; Gracitelli et al., 2014, 2015; Adhikari et al., 2015; Lei et al., 2015; Rukmini et al., 2015; Kelbsch et al., 2016; Najjar et al., 2018, 2021). By comparing protocols with different irradiance, duration, and wavelength, Adhikari et al. proposed that the PIPR could be measured using short-duration pulses (e.g., ≤ 1 s) with high melanopsin excitation and analyzed with 6-s metrics and/or plateau (Adhikari et al., 2015; Kelbsch et al., 2019). In Experiment 1, we evaluated the rod-weighted, cone-weighted, and ipRGC-weighted PLR features in patients with different stages of glaucoma by presenting different full-field stimuli through a binocular pupillometer to activate certain photoreceptor type-dominant responses. In rod-weighted PLR tests, a blue flash at 0.1 cd/m^2^ (wavelength 465 nm) was presented. In cone-weighted PLR tests, a blue field of 6 cd/m^2^ (wavelength 465 nm) was used to suppress rod activity for 2 min, followed by a red flash (10 cd/m^2^; wavelength 642 nm). The constriction amplitude, latency, and velocity of constriction and dilation were calculated in the rod/cone-weighted PLR tests. In the ipRGC-weighted PLR tests, a blue flash with a high luminance of 100 cd/m^2^ (wavelength 465 nm) was presented on the head-mounted screen. The 6-s PIPR and the abovementioned pupillometric parameters were calculated and evaluated as diagnostic indices for glaucoma.

During the progression of POAG, the loss of retinal nerve fibers and glaucomatous damage is often asymmetric. This asymmetry can be exploited in diagnostic tools for diagnosing patients with glaucoma (Chang et al., 2013; Hou et al., 2018). RAPDs can be diagnosed by comparing the pupil light response between both eyes with the SFT with/without neutral density filters using an indirect ophthalmoscope (Tatsumi et al., 2007; Schiefer et al., 2012). To eliminate shortcomings in terms of subjective bias and strict environmental conditions, computerized pupillometers have been used in several studies (Schiefer et al., 2012; Kelbsch et al., 2019; Temel et al., 2020). Volpe et al. (2000) proposed a portable, electronic, infrared pupillometer (Pupilscan II Type 9 Optical Unit) presenting the alternating light stimuli with true (23 milliwatts/cm^2^) or simulated (2 milliwatts/cm^2^) afferent pupillary defects. Stimulus cycles were set for alternating eye stimulations of 200 ms in duration every 1.2 s. Cohen et al. (2015) used the RAPDx pupillometer to elicit and record RAPDs. Nine pairs of bright white light stimuli (illuminance 36.81 lux) with a total cycle time for each stimulus of 2.1 s (0.3 s to estimate pupillary diameter, 0.1 s light on) were used. In our study, the RAPD test (Experiment 2) was induced by intermittent blue or red light flashes to increase the accuracy of the results (12 repetitions). The RAPD parameters were calculated and correlated with the structural and functional parameters. All PLR tests were carried out in a sitting position using the binocular pupillometer, introducing a portable, multipurpose diagnostic tool to assist in POAG diagnosis.

The visual function loss of POAG is mainly attributed to gradual and irreversible damage to the RGC axons in the optic nerve and thereby retrogradely causes degeneration of the RGC soma. However, clinical studies and histological studies have suggested that glaucomatous damage may not be limited to the inner retina (Nork et al., 2000; Fan et al., 2011). Nork et al. observed cone swelling in cadavers who had a clinical diagnosis of chronic glaucoma (Nork et al., 2000). Fan et al. found that glaucomatous damage may involve structural changes in the photoreceptor layer using OCT measurements (Fan et al., 2011). Kelbsch et al. (2016) detected an impairment of the synaptic pathway via rods and cones to both RGCs and ipRGCs through pupillary escape behavior (stimulus: 28 lux, 4 s red stimulus) in advanced POAG patients. In a cohort including 45 patients with POAG and 25 healthy control participants, Duque-Chica et al. (2018) found that PLR was significantly reduced under both 470 and 640 nm stimuli in patients with moderate and severe POAG, indicating a decrease in contributions from rods, cones, and intrinsically photosensitive retinal ganglion cells. Our data in Experiment 1 indicated a decreased constriction amplitude with slower constriction velocity in POAG patients in PLR induced by rod-weighted and cone-weighted stimuli. The constriction amplitude in cone-weighted PLR was highly correlated with retinal structural (e.g., C/D ratio, GCC thickness) and functional defects (MD score) of POAG. These findings further support the notion that glaucomatous damage may be more complex and that the outer retina may also be involved in the pathophysiological process of POAG.

In addition to rod and cone photoreceptors, ipRGCs also express the photopigment melanopsin (Berson et al., 2002; Barrionuevo et al., 2023). ipRGCs project to the suprachiasmatic nucleus (SCN) and the olivary pretectal nucleus (OPN), which are involved in circadian rhythm control and PLR (Chen et al., 2011). Several studies have proposed that ipRGCs are resistant to glaucomatous damage in experimental rodent models (Li et al., 2006; Honda et al., 2019). Obara et al. (2016) provided histologic evidence for reduced ipRGC density in the ganglion cell layer of retinas with severely staged glaucoma, but earlier alterations are not mentioned in this article. Rukmini et al. (2015) found that PLRs to high-irradiance blue light (469 nm) were more strongly associated with POAG severity than responses to red light (631 nm), with a significant linear correlation observed between pupil diameter and visual field MD score. Gracitelli et al. (2014) also showed that the MD score and RNFL thickness were positively associated with a sustained response to blue flashes (1 s, wavelength 470 nm, and luminance 250 cd/m^2^) in 76 eyes from 38 patients with primary open-angle glaucoma. Duque-Chica et al. (2018) found that the melanopsin-mediated pupil responses were not significantly different between patients with mild POAG and controls. While Najjar et al. (2018) observed reduced pupillary responses to moderate and high irradiances (11 log photons/cm^2^/s) of blue and red lights in patients with early-stage POAG, the alteration of PLR correlates with structural thinning of the RNFL, but not with visual field scores. Adhikari et al. (2016) observed ipRGC dysfunction in patients with early-stage glaucoma, preperimetric glaucoma, and suspected glaucoma using quadrant field stimulations. In Experiment 1, we used multiple parameters to assess the overall function of ipRGCs, including the constriction, dilation, and post-illumination variables. A reduction in pupillary constriction amplitudes and PIPR under the bright blue light stimulus was observed in POAG with different severities. In addition, we observed a slow recovery after constriction, and the delay of dilation was strongly correlated with SAP (MD score) and retinal structural defects (RNFL thickness, C/D ratio, cup volume, rim area, and GCC thickness), providing accurate diagnostic efficiency in POAG diagnosis. Other than the PIPR, time to max dilation in ipRGC-weighted stimulus (AUC = 0.866, sensitivity = 0.662, specificity = 0.913), constriction amplitude response to cone-weighted stimulus (AUC = 0.743, sensitivity = 0.696, specificity = 0.739), and constriction amplitude response to ipRGC-weighted stimuli (AUC = 0.713, sensitivity = 0.541, specificity = 0.826) also exhibited good diagnostic performance. Dilation latency or the combination of constriction amplitude, dilation latency, and PIPR could be considered as parameters to evaluate POAG severity.

Glaucoma damage is often more severe in one eye (Tatsumi et al., 2007; Hou et al., 2018). Inter-eye asymmetry can be used as a diagnostic tool for glaucoma (Hou et al., 2018). RAPD is a sign of unilateral or asymmetric damage of the anterior afferent visual pathways (Volpe et al., 2000). Prior studies demonstrated that an RAPD could be detectable in patients with glaucoma using an SFT, or pupillometry (Tatsumi et al., 2007; Hennessy et al., 2011; Schiefer et al., 2012; Chang et al., 2013; Charalel et al., 2014; Waisbourd et al., 2015; Pillai et al., 2019). However, the SFT requires considerable practice, and the RAPD could only be roughly estimated using the SFT test. In several studies, the SFT was carried out by manually presenting alternating white light stimuli. In a cohort including 29 glaucoma patients (chronic angle-closure glaucoma, steroid-induced glaucoma; and developmental glaucoma) with clinically detectable RAPD, Tatsumi et al. (2007) showed that the RNFL thickness in the more advanced eyes was on average reduced to ~73% of that in the less advanced eyes. In a study including 79 consecutive subjects with glaucomatous optic neuropathy in at least one eye, Schiefer et al. (2012) observed that an absolute value of RAPD of 0.3 log_10_ units or more was present in 25% of patients. Waisbourd et al. (2015) made use of the RAPDx pupillograph to evaluate the RAPD in 60 patients with glaucoma (including primary angle-closure glaucoma, normal tension glaucoma, and POAG) and 21 patients with ocular hypertension or glaucoma suspect. The mean asymmetry of MD was 4.43 ± 5.17 dB and that of RNFL was 9.91 ± 10.97 microns. The pupillography amplitude score was correlated with MD asymmetry (*r*^2^ = 0.41, *p* = 0.001) but not the RNFL asymmetry. In our study, we used binocular pupillometry and sequenced chromatic stimuli to better quantify the RAPD value. In Experiment 2, we found that the amplitude and duration of the anisocoria induced by intermittent chromatic light flashes were significantly larger in patients with POAG than in healthy controls. The inter-eye difference in the MD value was inversely correlated with the IPA value and anisocoria duration. The RAPD induced by intermittent blue flashes was more frequently observed in both controls and POAG patients. This result could be explained by the fact that the PLR to bright blue light was a combination of cone and ipRGCs, and the more post-illumination response was attributed to these cells. Consistent with previous studies, 52.7% of POAG patients exhibited RAPD, and 13.0% of healthy controls exhibited RAPD (blue flash induced) in our study (Chang et al., 2013; Charalel et al., 2014). This was probably due to the absence of RAPD in relatively symmetric glaucoma. The cumulative effect of a combination of IPA and anisocoria duration showed good performance in diagnosing asymmetric POAG. Using the binocular pupillometer, we were able to perform the full-field stimuli and the RAPD test in one sitting. To the best of our knowledge, this is the first study using alternating chromatic stimuli to measure RAPD test in POAG pupillometry studies. Binocular chromatic pupillometry is a portable, programmable vision monitoring and testing system for screening multiple ophthalmic diseases, including glaucoma.

One limitation of our study is that all the participants were dark-adapted shortly before stimuli; hence, rhodopsin was not entirely regenerated in rods to optimally capture light. The PLR to each stimulus could not be entirely isolated to a single photoreceptor type, and the response could only be considered as having one specific type with major participation (Kardon et al., 2009). In addition, we did not include preperimetric stage patients and patients with suspected POAG in our study; hence, the exact diagnostic accuracy could not be obtained. Further screening with a large sample should be carried out to improve the accuracy of glaucoma detection.

In this study, we provide proof of concept for the use of binocular chromatic pupillometry for identifying POAG with a strong correlation to retinal structural measurements and visual functional impairment. This could be especially useful for patients who are unable to use the chinrest or for children. Binocular chromatic pupillometry could accurately and rapidly detect glaucomatous damage. The low-cost and patient-friendly program could be used to screen for multiple retinal and optic neuropathies in population-based screening.

## Data availability statement

The original contributions presented in the study are included in the article/supplementary material, further inquiries can be directed to the corresponding author.

## Ethics statement

The studies involving human participants were reviewed and approved by the human subjects review committee of the Eye and ENT Hospital of Fudan University. The patients/participants provided their written informed consent to participate in this study.

## Author contributions

XSu and YQ: conceptualization. YQ, HD, ZZ, YS, and JH: methodology. YQ, ZZ, and RL: data analysis. YQ and XSu: writing—original draft preparation. TL, TZ, JW, GZ, XSo, and YZ: writing—review and editing. XSu and GZ: funding acquisition. All authors contributed to the article and approved the submitted version.
